# A direct endoscopic approach for left-sided infrarenal para-aortic lymphadenectomy immediately after hysterectomy for endometrial cancer treatment: left dome formation (LDF)

**DOI:** 10.1007/s00464-019-07103-3

**Published:** 2019-09-05

**Authors:** Yasunari Mizumoto, Junpei Iwadare, Kyohei Nakade, Takeshi Obata, Takeo Matsumoto, Kyosuke Kagami, Takashi Iizuka, Ayumi Matsuoka, Masanori Ono, Mitsuhiro Nakamura, Hiroshi Fujiwara

**Affiliations:** Department of Obstetrics and Gynecology, Kanazawa University Graduate School of Medicine, 13-1 Takaramachi, Kanazawa, Ishikawa 920-8641 Japan

**Keywords:** Left-sided infrarenal para-aortic lymphadenectomy, Direct access, Sentinel node sampling, Dome formation, Endometrial cancer

## Abstract

**Background:**

Endoscopic surgery for infrarenal para-aortic lymphadenectomy has been widely accepted. Two major approaches, “transperitoneal” and “extraperitoneal”, are generally used; however, they have several disadvantages. A “transperitoneal” approach to the left para-aortic region is usually indirect, often performed after wide extension of the right para-aortic region. An “extraperitoneal” approach is unsuitable when a peritoneal tear exists after a prior surgical procedure such as hysterectomy. Here, we propose a modified transperitoneal technique, “Left dome formation (LDF),” which directly provides a surgical field for left infrarenal para-aortic lymphadenectomy even after hysterectomy.

**Methods:**

The LDF procedure comprised three processes: (1) setting, (2) dissection of inframesenteric lymph nodes (step 1), and (3) dissection of infrarenal lymph nodes (step 2). Setting: two trocars were added 4 cm bilateral to the low-mid abdominal trocar that was used in prior hysterectomy. Step 1: The posterior layer of the renal fascia along with the left ureter and left ovarian vessel were separated from the left common iliac artery and iliopsoas. Left inframesentric nodes were removed from the surgical field. Step 2: The left ureter was isolated from the posterior renal fascia, and the dome was expanded cranially to the left renal vein, with the ovarian vein always visualizable at the dome ceiling. Left infrarenal nodes were removed.

**Results:**

We applied LDF to ten endometrial cancer patients, recommended for additional dissection of para-aortic nodes based on intraoperative evaluation using the laparoscopically removed uterus. The operative time and number of removed lymph nodes in Step 1 and Step 2 were 28.8 (20–49) min and 5.3 (2–10) and 54.6 (52–70) min and 6.5 (1–11), respectively. Blood loss was below 50 ml. No serious organ injury occurred during procedures.

**Conclusion:**

Since the left ureter is always observable, LDF procedure facilitates effective surgery to overcome the anatomical complexity of the left para-aortic region and is potentially useful for sentinel node sampling.

Infrarenal para-aortic lymphadenectomy is an essential technique for surgical staging and treatment, and endoscopic procedures have been accepted and applied for gynecological malignancies. The techniques that are well described and widely used are the “Transperitoneal technique” [1] and “Extraperitoneal technique” [2]. Although both techniques are safe and effective, they have several disadvantages in certain situations. To overcome the anatomical complexity of the left-sided para-aortic region, composed of the descending mesocolon, Toldt’s fusion fascia, Gerota’s fascia, and the posterior renal fascia, the “Transperitoneal technique” is often used subsequent to right-sided para-aortic lymphadenectomy [1]. Consequently, this procedure is unsuitable for direct sampling of left-sided para-aortic lymph nodes. In contrast, the “Extraperitoneal technique” enables to firstly reach the left-sided para-aortic region. However, it secures the surgical field of view by filling the retroperitoneal cavity with carbon dioxide gas, but this procedure needs to be performed within an intact peritoneum. Accordingly, it is impossible to perform this technique directly after intraoperative evaluation of pathological risk factors using the removed uterus. Based on this background, we developed a novel modified transperitoneal procedure, “Left dome formation (LDF),” which enables to directly reach the left-sided para-aortic region even in the presence of a peritoneal tear after hysterectomy or salpingo-oophorectomy. Here, we describe the technique and outcome of the LDF procedure and discuss its strengths and limitations.

## Materials and methods

To date, we have applied the LDF procedure to women with endometrial cancer, who were recommended to undergo additional dissection of para-aortic lymph nodes based on the intraoperative evaluation of risk factors for metastasis using the laparoscopically removed uterus. The LDF procedure consisted of three processes: (1) setting, (2) dissection of inframesenteric lymph nodes (step 1), and (3) dissection of infrarenal lymph nodes (step 2). This study was approved by Kanazawa University Ethics Committee (No. 2848.)

### Setting

**Fig. 1:**
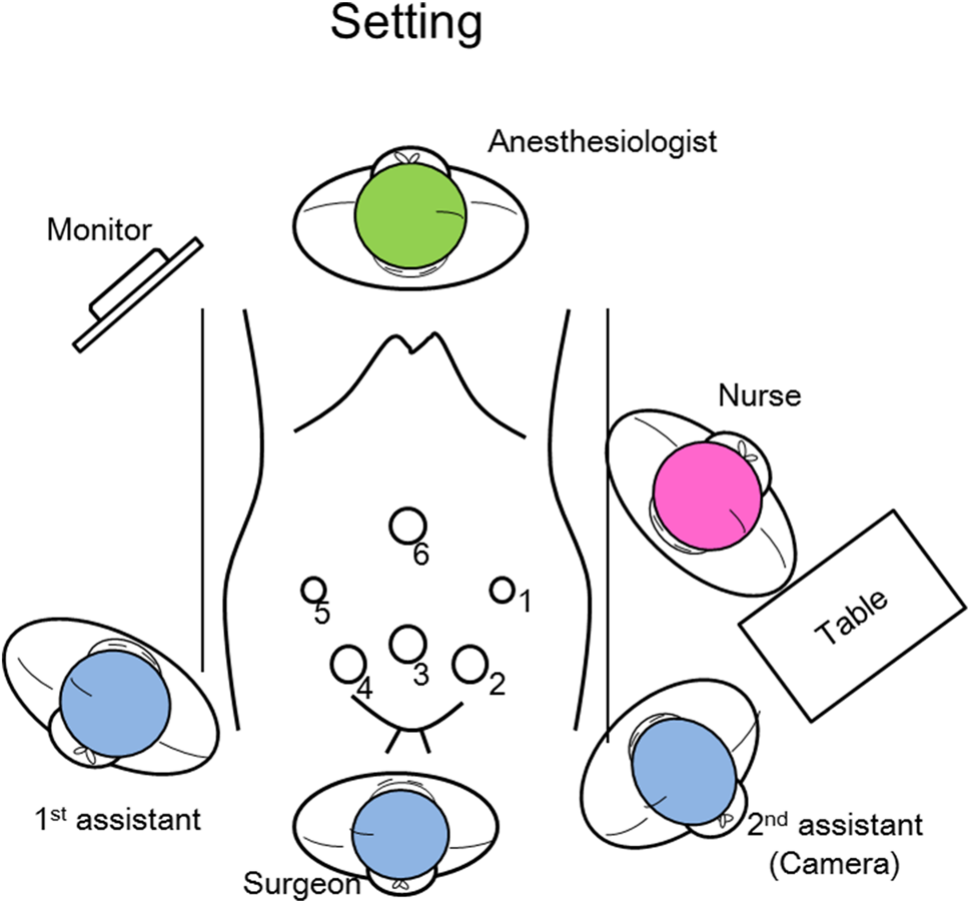
Setting for left dome formation procedure. A lithotomy position with 0°–10° head-down. Trocar Nos. 1, 3, 5, and 6 are introduced for hysterectomy, bilateral salpingo-oophorectomy, and pelvic lymphadenectomy. Trocar Nos. 2 and 4 are added for the LDF procedure. The surgeon uses Nos. 1 and 3 for the procedure, No. 2 for the camera, and No. 4 for the endoretractor

The patient was put in a lithotomy position with slight head-down (0°–10°). The surgeon stood between the legs, while the first assistant stood on the right side and the second assistant who held the camera stood on the left side of the patient. The surgeon and assistants used the monitor on the right cranial side of the patient (Fig. 1). Prior to para-aortic lymphadenectomy, hysterectomy and pelvic lymphadenectomy were performed with the following: 12-mm trocar for camera-holding at the umbilicus (Fig. 1, circle 6), 12-mm trocar at the mid-low abdomen 4 cm cephalad to the symphysis pubis (Fig. 1, circle 3), and two 5-mm trocars 3 cm medial to each side of the anterior iliac crest (Fig. 1, circles 1 and 5). To perform para-aortic lymph node sampling/dissection, two additional 12-mm trocars were introduced 4 cm lateral to the mid lower abdominal 12-mm trocar (Fig. 1, circles 2 and 4). The ENDOEYE FLEX 3D (OLYMPUS) scope, which has the advantages of 3D vision and a flexible camera head, was used for this operation.

### Step 1

**Fig. 2:**
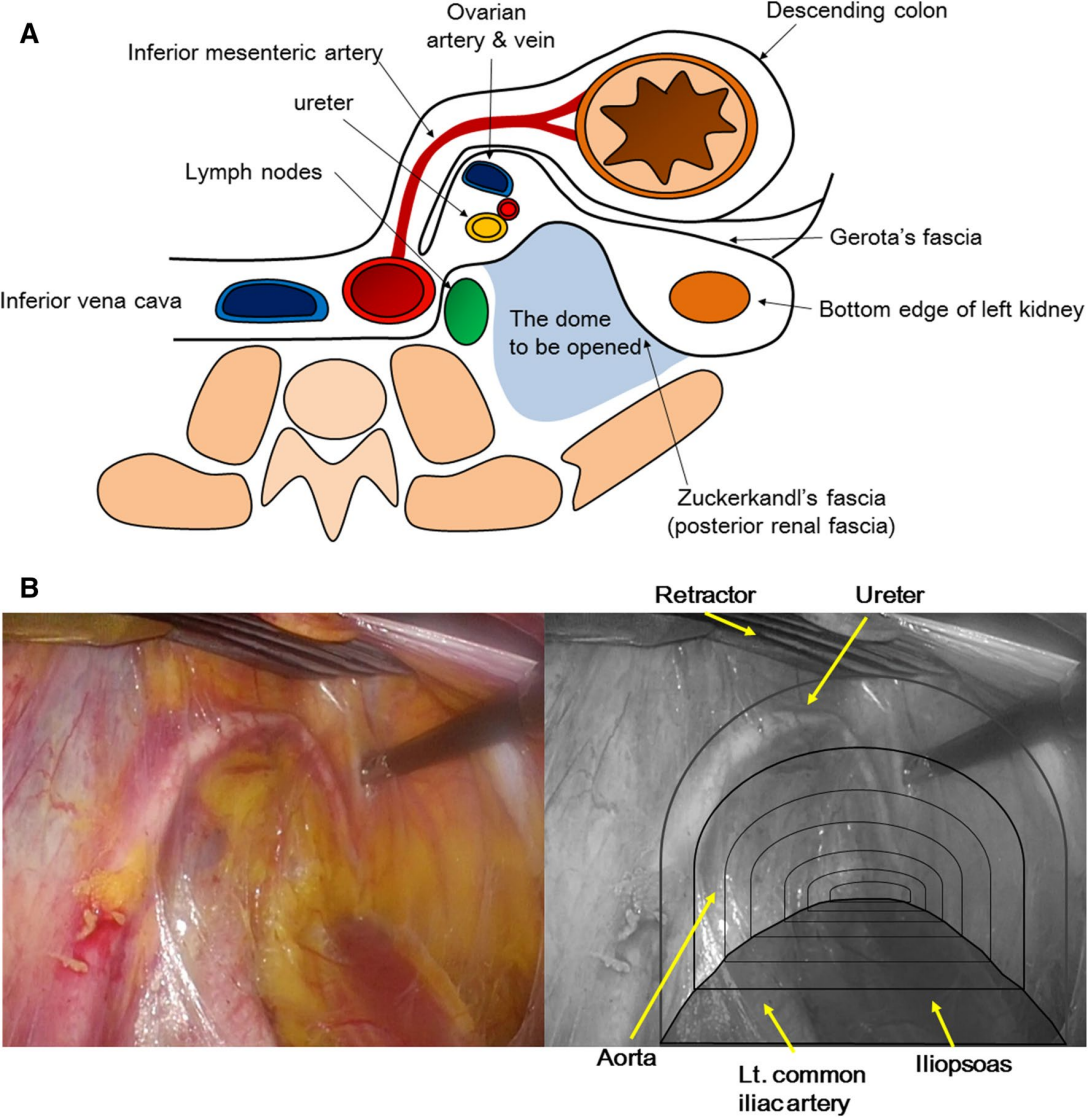
Step 1: Development of the dome for inframesenteric para-aortic lymphadenectomy. **A** Schema of anatomy for development of inframesenteric region. The posterior renal fascia is separated from the left common iliac artery and iliopsoas and forms the ceiling of the dome. **B** Surgical view of the dome. The ureter is visualized in the ceiling of the dome

Using an additional left 12-mm trocar (circle 2) as a camera port, the left ureter identified medio-dorsal to the infundibulopelvic ligament, which was dissected during prior salpingo-oophorectomy, was raised up along with the posterior renal fascia, also known as Zuckerkandl’s fascia [3] and gently separated from the left common iliac artery and fascia of the iliopsoas muscle. By expanding the space further cranially, the surgical field was prepared up to 2 cm superior to the origin of the inferior mesenteric artery, just like forming a dome. Seen through the monitor, the first dome is composed of Zuckerkandl’s fascia with the ureter in sight at the ceiling, iliopsoas on the floor, and the beating aorta is identified on the left side of the dome. The ceiling of the dome was held with Endo Retract II (Covidien) in order to keep the surgical field secure (Fig. 2A, B).

### Step 2

**Fig. 3:**
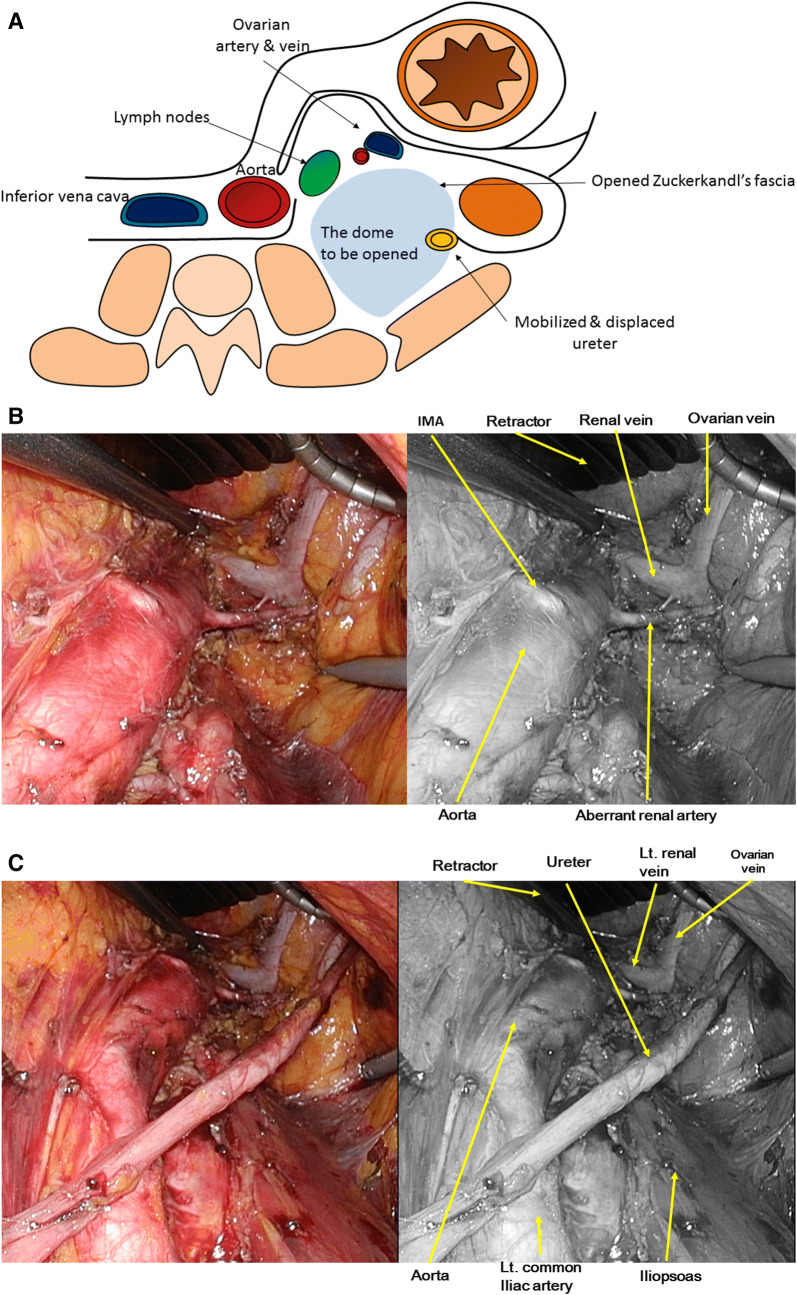
Step 2: Development of the dome for infrarenal para-aortic lymphadenectomy. **A** Schema of anatomy for the development of infrarenal para-aortic lymphadenectomy. The posterior renal facia is opened and the ureter is isolated dorso-laterally. **B** Surgical view after lymphadenectomy. The ovarian vein and connecting renal vein are visualized. The aberrant renal artery is also skeletonized safely

By separating and dislocating the left ureter from the ceiling of the dome toward the left kidney, the space between Gerota’s fascia and Zuckerkandl’s fascia was opened. The dome is further expanded cranially, while always keeping the ovarian vein on the ceiling of the dome in view, up to the level around the anastomotic site of the ovarian vein to the left renal vein. Seen through the monitor, the secondary extended dome is composed of the aorta on the left, Gerota’s fascia with the left ovarian vein at the ceiling, kidney on the right, fascia of the iliopsoas on the floor, and left renal vein at the dead end (Fig. 3A, B). Maintaining the surgical field with Endo Retract II, lymph node dissection was performed using a vessel-sealing system with mono- and bipolar devices.

### Additional step

After left infrarenal para-aortic lymph node dissection, using a right 12-mm trocar (circle 4) as a camera port, a retroperitoneal surgical view of the right para-aortic region was then constructed by elevating the posterior layer of the right renal fascia along with the right ureter and ovarian vessels to form the right dome. After sealing of the right ovarian vessels near anastomotic sites, routine right infrarenal para-aortic and pericaval lymphadenectomy was performed through this dome formation.

## Results

Until August 2018, we applied the LDF procedure to ten endometrial cancer patients. Characteristics of the patients are presented in Table 1. Briefly, in nearly all cases, the pre-operative diagnosis by histological pathology and MRI was endometrioid carcinoma grade 1 or 2 without deep myometrial invasion, which corresponds to FIGO Stage IA. However, frozen section analysis overturned this diagnosis, and additional para-aortic lymph node dissection was carried out. The median BMI was 22.3, with a range of 19.8 to 25.4. The median operative durations for extension of the left inframesenteric area in step 1 and left infrarenal area in step 2 were 16.7 (range 10–26) and 12.1 (range 6–26) min, respectively. The median operative durations for lymph node dissection of the left inframesenteric and left infrarenal areas were 20.2 (range 11–28) and 34.4 (range 22–46) min, respectively. The mean total operative duration for left para-aortic lymphadenectomy was 83 (range 77–114) min. The mean numbers of dissected lymph nodes from the left inframesenteric and left infrarenal areas were 5.3 (2–10) and 6.5 (1–11), respectively. When the data of the right infrarenal para-aortic lymphadenectomy procedure, as described in additional step, were included, the total operative duration for infrarenal para-aortic lymphadenectomy was 189.4 (179–236) min, and the total number of harvested lymph nodes was 24.7 (9–44).In all cases, blood loss during the procedure was < 50 ml (data not shown). No major intraoperative complication such as vascular, bowel, or ureteral injury was encountered during the procedure. One case of port-site hernia (Fig. 1, circle 2) and one case of chylous ascites occurred after surgery (Table 2).

**Table 1:** Patients’ characteristics

Patiens’ characteristics (*N* = 10)	
Age (range)	62.2 (47–80)
BMI	22.3 (19.8–25.4)
Pre-operative evaluation	
MI < 1/2 (MRI)	9/10
MI > 1/2 (MRI)	1/10
Endometrioid grade 1	5/10
Grade 2	4/10
Grade 3	1/10
Frozen section diagnosis	
MI > 1/2	5/10
Endometrioid grade 3	4/10
FIGO staging (2008)	
IA	6/10
IB	2/10
IIIA/IIIC	2/10
Complication	
Port-site hernia, ileus	1/10
Chylous ascites	1/10

**Table 2:** Outcome of left dome formation procedure

Lymph node dissection (*N* = 10)	Time (min)		Mean LNs removed (range)
	Dome formation	Lymph node removal	
Left inframesenteric area	16.7 (10–26)	20.2 (11–28)	5.3 (2–10)
Left infrarenal area	12.1 (5–25)	34.4 (22–46)	6.5 (1–11)
Left para-aortic area	28.8 (20–49)	54.6 (52–70)	11.8 (5–21)

## Discussion

In this study, we demonstrated a novel direct endoscopic approach for left-sided infrarenal para-aortic lymph node dissection, “Left dome formation (LDF).” This procedure has three main strengths. First, LDF can be used to develop a surgical field with visual confirmation of the left ureter and gonadal vessels, which allows surgeons to master this new method safely and may possibly contribute to the shorter surgical duration. Second, LDF can be implemented even when the retroperitoneal space is widely opened, which allows surgeons to perform additional infrarenal para-aortic lymph node sampling/dissection after hysterectomy or oophorectomy. Third, LDF allows to directly reach the left para-aortic area, enabling surgeons to select this method for sentinel infrarenal para-aortic lymph node sampling.

There are two major approaches for para-aortic lymphadenectomy in gynecologic endoscopic surgery: trans- and extraperitoneal approaches. In general, the transperitoneal approach is initiated by opening the peritoneum near the right common iliac artery, followed by passing through the descending mesocolon, Toldt’s fusion fascia, Georta’s fascia, and Zuckerkandl’s fascia to reach the left para-aortic area. During this procedure, it is sometimes difficult to identify the inferior mesenteric artery, left ureter, or gonadal vessels due to the complexity of the surrounding anatomy. Furthermore, it is even more difficult when aberrant vessels exist. To reduce the risk of left ureteral or vascular injury, initial clearing of the surgical field through right para-aortic and pericaval lymphadenectomy is preferentially performed prior to the opening of the retroperitoneal space around the left para-aortic area. In contrast, we can directly reach the left para-aortic area by the LDF procedure, easily identifying the left ureter and gonadal vessels. This advantage may be critical, especially when sampling of a suspected lymph node in the left area is mandatory. On the other hand, a retroperitoneal approach directly provides a favorable surgical field involving the left para-aortic area for lymph node dissection. However, we cannot select this procedure when the peritoneum is widely opened after hysterectomy or oophorectomy because the intact peritoneum is essential to keep the surgical field secure by filling with carbon dioxide gas.

Although complete staging surgery for endometrial cancer includes hysterectomy, bilateral salpingo-oophorectomy, and pelvic/para-aortic lymph node dissection, less invasive surgery by omitting lymphadenectomy is currently permitted when the tumor is considered to show a low risk of recurrence [4]. Accordingly, pre-surgical evaluation of the current risk factors for recurrence including deep myometrial invasion, histologically high-grade tumors, and lymph node metastasis is important. Although the pre-operative diagnosis by histological pathology of a biopsied specimen and MRI is accurate in most cases, frozen section evaluation of the removed uterus or lymph node may overturn the diagnosis in certain cases [5], where additional regional lymphadenectomy is recommended. Considering the advantages of our new method, it is reasonable to apply LDF to the above cases.

The efficacy of surgical staging with sentinel lymph node (SLN) mapping and ultrastaging of the sentinel lymph node is under evaluation in developed countries. When an indocyanine green tracer was injected into the uterine cervix, isolated para-aortic sentinel lymph nodes were detected in 1% of patients in the FIRES trial [6]. It was also reported that the ultrastaging of sentinel lymph nodes revealed relatively high positive nodes in low-risk or grade 1 endometrial cancer, although the prognostic importance needs to be elucidated. Based on this background, since the opening order of right-and-left sides of domes (step 1 and an additional step) can be freely selected, we consider that LDF will become one of the preferred procedures for infrarenal para-aortic lymph node sampling.

This study has some limitations. Unintentionally, the cases recruited in this study (mean BMI: 22.3 kg/m^2^) did not include obese women. Obesity may be one of the important limitations of our dome formation method, because excess fat on the mesocolon may hamper maintenance of the dome opened by retractors, especially in the infrarenal region. Another possible limitation is that complete dissection or passage of a retro-aortic and/or retro-caval lymph node is relatively difficult, being similar to the transperitoneal approach or open surgery.

In conclusion, we propose a novel technique for left-sided para-aortic lymph node sampling/dissection. This procedure is characterized by unilateral dome formation and has several advantages: (1) safe preparation of a surgical field with visual confirmation of the running of the left ureter and gonadal vessels, (2) possible application immediately after hysterectomy or oophorectomy, (3) free selection of the opening order of right-and-left retroperitoneal spaces around para-aortic regions. Since this method is simple, LDF may become one of the standard methods for infrarenal para-aortic lymph node sampling/dissection by gynecologic oncologists.
